# Neonatal septicemia caused by a rare pathogen: Raoultella planticola - a report of four cases

**DOI:** 10.1186/s12879-020-05409-5

**Published:** 2020-09-16

**Authors:** Xianrui Chen, Shaoqing Guo, Dengli Liu, Meizhen Zhong

**Affiliations:** 1 Department of Pediatrics, The First Affiliated Hospital of Xiamen University, No.55 Zhenhai Road, Xiamen, 361003 China; 2 Pediatric Key Laboratory of Xiamen, No.55 Zhenhai Road, Xiamen, 361003 China; 3 Institute of Pediatrics, School of Medicine, Xiamen University, No.55 Zhenhai Road, Xiamen, 361003 China

**Keywords:** Newborn;septicemia, Raoultella planticola, Nosocomial infections, Case report

## Abstract

**Background:**

Raoultella planticola(R.planticola) is a very rare opportunistic pathogen and sometimes even associated with fatal infection in pediatric cases. Recently,the emergence of carbapenem resistance strains are constantly being reported and a growing source of concern for pediatricians.

**Case presentation:**

We reported 4 cases of neonatal septicemia caused by Raoultella planticola. Their gestational age was 211 to 269 days, and their birth weight was 1490 to 3000 g.The R. planticola infections were detected on the 9th to 27th day after hospitalization and occured between May and June. They clinically manifested as poor mental response, recurrent cyanosis, apnea, decreased heart rate and blood oxygen, recurrent jaundice, fever or nonelevation of body temperature. The C-reactive protein and procalcitonin were elevated at significantly in the initial phase of the infection,and they had leukocytosis or leukopenia. Prior to R.planticola infection,all of them recevied at least one broad-spectrum antibiotic for 7-27d.All the R.planticola strains detected were only sensitive to amikacin, but resistant to other groups of drugs: cephalosporins (such as cefazolin, ceftetan,etc) and penicillins (such as ampicillin-sulbactam,piperacillin,etc),and even developed resistance to carbapenem. All the infants were clinically cured and discharged with overall good prognosis.

**Conclusion:**

Neonatal septicemia caused by Raoultella planticola mostly occured in hot and humid summer, which lack specific clinical manifestations. Pediatricians should keep in mind that R. planticola can be a potential source of neonatal sepsis and even has the potential to acquire carbapenem-resistance. Preventing outbreaks of epidemics requires early detection, timely diagnosis and treatment, and active isolation.

## Background

Raoultella planticola(R.planticola) is an oxygen-demanding, membrane-free, powerless Gram-negative *E. coli* [1],including three known species of lyophilized Raoultella, ornine raoulella and earthen raoulella according to the rRNA and rpoB sequences in 200 1[2]. R.planticola is found in the natural environment such as water, soil and plants, etc. It also can exist in animals mucous membrane, but it is rarely isolated from clinical specimens. In recent years,there were some literature been reported that R.planticola caused different infections (bacteremia, pneumonia, retroperitoneal abscess,cellulitis,cholangitis and soft tissue infection) in adults and children. In 2018,the first case of Raoultella planticola septicemia suspected secondary to bilateral conjunctivitis in a infant was successfully treated with piperacillin-tarzobatam [3]. In this study,4 cases of neonatal septicaemia caused by Raoultella planticol were retrospectively reviewed from an electronic database of our hospital,so as to characterize its risk factors, clinical features and antimicrobial susceptibility. We hope our works is helpful to understand the neonatal R. planticola infections for pediatricians.

## Case presentation

Between July 2014 and July 2019, 13,995 cases were admitted to the Neonatal Intensive Care Unit in our hospital, of which 281 cases were diagnosed as sepsis and 4 episodes of R.planticola were detected from blood culture specimens. In the same period, we did not find Raoultella planticola isolates from different clinical specimens,which included 1890 samples of other body fluids (such as sputum, cerebrospinal fluid, broncho-alveolar lavage fluid, pleural effusion, abdominal effusion, secretion or exudate, etc.) and catheter (such as PICC, tracheal catheter, thoracic drainage tube, abdominal drainage tube, etc.)culture. The clinical data on 4 cases was shown in Tables 1 and 2.

**Table 1:** General conditions of 4 cases

Patient	Gender	Gestational age	Birth weight	Maternal illness	Fertilization way	Inpatient days	Delivery way	Twins or multiples	Congenital malformations	The date of isolation of R. planticola
1	male	211	1490	/	natural pregnancy	45	Cesarean delivery	single birth	Patent foramen ovale	13/06/2019
2	male	253	1910	/	in vitro fertilization	68	Cesarean delivery	twins	Patent foramen ovale	17/06/2019
3	female	215	1700	Gestational diabetes	natural pregnancy	41	Transvaginal delivery	single birth	Patent ductus arteriosus	28/05/2015
4	female	269	3000	/	natural pregnancy	20	Transvaginal delivery	single birth	lipoma	24/05/2015

**Table 2:** Clinical manifestations in 4 cases

Patient	Blood culture time (PND)	Nasal feeding time	Mechanical ventilation time	nCPAP	Oxygen time	antibiotics before infection	antibiotics after infection
1	26th	39	4	25	39	Piperacillin tazobartan sodium (9d + 3d)	Cefepime (14d)
						Meropenem (7d + 3d)	
2	27th	35	7	14	28	Piperacillin sodium tazobartan (8d), Cefoperazone/Sulbactam (4d), mepin (9d), vancomycin (4d), cefotaxime sodium (7d)	Cefoperazone/Sulbactam(2d) imipenem (21d) meropenem (14d), amphotericin B(10d)
						Fluconazole (3d), amphotericin B(18d)	
3	20th	26	3	5	21	Cefotaxime (14d)	Meropenem (14d), piperacillin tazobartan (7d), benzacillin (7d) fluconazole (10d)
						Cefoperazone sulbactam (3d)	
4	9th	11	6	5	13	Piperacillin / Tazobactam (7d)	Meropenem (14d)

*PND* Postnatal day, *nCPAP* Nasal Continuous positive airway pressure,d day

### Case 1

A 30-gestional week-old male infant weighing 1430 g was born to a mother for threatened preterm delivery. After 18 days of nasal Continuous positive airway pressure (nCPAP),High flow nasal cannula (HFNC) was used for respiratory support in the baby. On the 26th postnatal day (PND), poor feeding,cyanosis, apnea,desaturation,fever (38.7 °C) and decreased muscle tone were observed, he was intubated. After presenting symptoms of sepsis, meropenam was empirically applied for treatment, but the temperature was repeated. According to antibiotic susceptibility tests on the 29th PND, he completed 14 days of cefepime antibiotic therapy with clinical improvement and was extubated on the 32nd PND.

### Case 2

A male infant with gestational age of 36 weeks and a birth weight of 1910 g was born to a mother for threatened preterm delivery. The use of nCPAP or HFNC as respiratory support in the infant was until neonatal sepsis occurred on the 27th PND. When cyanosis,desaturation,fever (39.8 °C) and tachycardia were observed, he who did require mechanical ventilation was intubated. After Imipenem was initiated,clinical manifestations of neonatal sepsis improved within 48 h. Although the blood culture was positive for R. planticola which was resistant to Imipenem, the use of Imipenem therapy for 21 days made him full recovery.

### Case 3

A 30-gestional week-old female infant weighing 1700 g was born by vaginal delivery. NCPAP and nasal cannulas are used to provide oxygen support for the infant in the neonatal intensive care unit (NICU). She received cefotaxime for 14 days and cefoperazone sulbactam for 3 days as antibiotic treatment for neonatal pneumonia. On the 20th PND,lethargy,cyanosis,desaturation and decreasing body temperature were suspected neonatal early-onset sepsis (EOS), treating it required intubation and mechanical ventilation with meropenam empirically applied. She started to resolve on the 2nd day of antibiotherapy and antimicrobial susceptibility testing finally confirmed the R.planticola strains susceptible to meropenem.

### Case 4

A female full-term infant with a birth weight of 3000 g was was admitted to our hospital due to neonatal jaundice and lethargy. On the second PND, she was intubated and received mechanical ventilation when neonatal respiratory distress syndrome occurred. And chest radiographs further proved neonatal pneumonia and pulmonary hemorrhage. After 7 days duration of Piperacillin/ Tazobactam as therapy for Neonatal Pneumonia,she manifested as hyperthermia and tachycardia with leukocytosis (52,510/mm3),elevated C-reactive protein (76 mg/L) and procalcitonin (13.8 g/L) levels. Considering the neonatal sepsis, empirical meropenem were started. The R. planticola was reported as the same antibiogram from the blood culture. She made full recovery completing a 14-day treatment course.

All the patients received at least broad-spectrum antibiotics prior to the occurrence of neonatal septicemia caused by R.planticola. Meanwhile,they were in clinical convalescence of pneumonia, without mechanical ventilation for more than 1 week (7–18 days). Cerebrospinal fluid (CSF) analysis in all infants were normal when sepsis occurred. And they never did not receive peripheral central intravenous tube (PICC) and umbilical venous tube. The interval of R.planticola bacteremia in case 1,2 and case 3,4 was more than 4 years, but they occured in the summer from May to June. Raoultella planticola was isolated from blood culture samples in all patients showed varying degrees of drug resistance. All strains were sensitive to amikacin, and that isolated from case 1, 2 and 3 were sensitive to cotrimoxazole. Although cefepime was sensitive in case 1 and 2, it was resistant in case 3 and case 4.In addition, cases 3 and 4 were sensitive to ciprofloxacin, gentamicin, meropenem, levofloxacin and furantoin. Only case 2 was sensitive to piperacillin/tazobactam. All infants were cured and discharged. The clinical data on the four cases are shown in Table 3. The susceptibility testing results are shown in Table 4.

**Table 3:** Laboratory test results in 4 cases

Patient	WBC (10^9/L)	NE%	CRP (mg/L)	HGB (g/L)	PLT (10^9/L)	PCT (ng/mL)	G test	Number of positive blood culture	Other bacterial infections
1	18.06	89.7	13.54	123	146	15.13	negative	1	/
2	22.23	74	110.16	116	65	12.90	negative	3	Candida gigas, *Klebsiella pneumoniae*
3	1.89	16.90	8	101	125	/	negative	1	/
4	52.51	89.40	76	124	532	13.8	negative	1	/

*WBC* White blood cell count, *CRP* C-reactive protein, NE% neutrophil percentage, *HGB* Hemoglobin, *PLT* Blood platelet count, *PCT* Procaicltonin, G test (1,3) beta-D-glucan assay

**Table 4:** Antimicrobial susceptibility

antibiotics	Patient 1		Patient 2		Patient 3		Patient 4	
	antibiotic susceptibility	MIC	antibiotic susceptibility	MIC	antibiotic susceptibility	MIC	antibiotic susceptibility	MIC
Amikacin	S	<=2.0	S	<=2.0	S	<=2.0	S	<=2.0
Ampicillin-sulbactam	R	> = 32.0	R	> = 32.0	R	> = 32.0	R	> = 32.0
Aztreonam	R	16	R	16	R	16	R	16
Cefazolin	R	> = 64.0	R	> = 64.0	R	> = 64.0	R	> = 64.0
Cefepime	S	2	S	2	R	16	R	16
Cefotetan	R	> = 64.0	R	> = 64.0	R	> = 64.0	R	> = 64.0
Ceftazidime	R	> = 64.0	R	> = 64.0	R	> = 64.0	R	> = 64.0
Ceftriaxone	R	> = 64.0	R	32	R	> = 64.0	R	> = 64.0
Cefuroxime	R	> = 64.0	R	> = 64.0	R	> = 64.0	R	> = 64.0
Cefuroxime Axetil	R	> = 64.0	R	> = 64.0	R	> = 64.0	R	> = 64.0
Ciprofloxacin	R	1	R	1	S	<=0.25	S	<=0.25
Gentamicin	R	> = 16.0	R	> = 16.0	S	<=1	S	<=1.0
Levofloxacin	R	4	I	1	S	<=0.25	S	<=0.25
Piperacillin	R	> = 128.0	R	> = 128.0	R	> = 128.0	R	> = 128.0
Piperacillin/Tazobactam	I	32	S	16	I	64	I	64
Tobramycin	R	> = 16.0	I	8	S	4	S	4
Trimethoprim/sulfamethoxazole	S	<=20.0	S	<=20.0	S	<=20	R	> = 320
Imipenem	R	> = 16.0	R	8	R	8	R	4
Meropenem	I	> = 16.0	R	> = 16.0	S	4	S	4

*MIC* Minimum inhibitory concentration, *S* Sensitive, *R* Resistance, *I* Intermediary

## Discussion and conclusions

Although the survival rate of premature infants has been significantly improved in recent years, neonatal sepsis is still one of the main causes of neonatal death, especially for premature infants with small gestational age and low birth weight [4]. The pathogen distribution of neonatal sepsis varies with age, region, and even NICU. R. planticola is a rare cause of clinical infection in children and especially in newborns, it has been recognized as an emerging threat. Early and precise identification of R.planticola infection are very important to improve the prognosis of premature infants and to control the spread of this bacteria.

In this paper, the overall rate of nosocomial infection associated with neonatal sepsis was was 0.02% (281/13995), the R.planticola infection is responsible for 1.4%(4/281) of nosocomial infection in our NICU. Demiray T et al. [5] reported that R. planticola constituted 0.40% (*n* = 42) of all infectious agents from more than 1000 culture samples,and the incidence of R.planticola infections were identified as high as 80.9% (34/42 )[5], especially in ICU. Furthermore,the results demonstrated that the rate of R.planticola infection was a gradual increase year by year, and even if there was no epidemic at present, it should be paid enough attention by clinicians. R.planticola generally lives in the external environment, but may also exist in the hospital environment, and cause water pollution. In 2014, Garcia-San et al. [6] reported that a batch of liquid soap for hospital use was contaminated by R.planticola, but fortunately there was no outbreak of epidemic infection.

In our cases, all infants received at least one broad-spectrum antibiotic for more than 7 days before R.planticola infection, which indicated that infants with low immunity or potential diseases were susceptible to infection. R.planticola infections in different patterns (such as sepsis, biliary tract infection, pelvic cellulitis, urinary tract infection, etc.) seemed to occur mainly in immunocompromised patients [1, 7, 8]. Chun et al. [9] retrospectively reviewed 20 R.planticola bacteremia cases with a nosocomial infection rate of 0.08%(20/26208) and found that of the 17 (85%)patients had underlying malignant conditions. In China, Li Gang et al .[10] also performed a retrospective review of the clinical distribution and drug-resistance patterns of 34 cases with R.planticola bacteremia and the majority of these patients had immunologic functions low or the flaw.

All our patients received ventilator-assisted ventilation for more than 1 week before R.planticola infection,which suggested that tracheal intubation mechanical ventilation may also be a risk factor for infection. However, no invasive peripheral venous tube and umbilical venous tube surgery in our cases were performed, it was inconsistent with some previously researches suggesting that invasive operations may provide an infection pathway [11]. The clinical manifestations of R. planticola infections are similar to other bacterial infections. In our study, 4 patients presented poor feeding,cyanosis, apnea,desaturation,tachycardia and lethargy after R. planticola bacteremia occured. Prior to R. planticola bacteremia, Case 2 had repeatedly hyperpyrexia due to *Candida guilliermondii* infection. As a consequence,the clinical features of neonatal sepsis by R. planticola infections lack specificity. All infants had no intracranial infection and this was consistent with the published reportes [1, 3, 9].

There were significant differences in drug resistance of R.planticola strains in 4 cases. For example, R.planticola from blood stream infections in cases 3 and 4 were sensitive to ciprofloxacin, gentamicin and cefepime,while cases 1 and 2 were resistant to them. We determined the multidrug-resistant R. planticola isolates,which were resistant to such as cefazolin,ceftetan,ampicillin/sulbactam, piperacillin,imipenem,etc. It is significantly different from the research results of li gang et al. [10], which showed that no carbapene-resistant isolates or multidrug-resistant isolates have been determined in 34 strains of R.planticola derived from sputum or tracheal secretions. According to susceptibility testing,most of R.planticola were resistant to ampicillin, cefuroxime, cefuroxime ester and piperacillin, while carbapenem and aminoglycosides compounds were still the most effective antimicrobial agents [5]. In 2019, Pacilli M et al .[12] systematically reviewed the literature about R.planticola bacteremia in children and found that R.planticola had a good sensitivity to all antibiotics except ampicillin. However,there were some data about the carbapenem-resistant isolates of R.planticola [13, 14], of which the rate of resistance to melopinan, ertaxpenem and amine peruminis is 7.1% (3/42) [5]. As more and more multidrug-resistant isolates of R.planticola were identified in different hospitals of the world,especially carbapenem-resistant R.planticola,it was presumed that these infections may be associated with the abuse of clinical antimicrobials [15]. In the future, carbapenem-resistant R.planticola may bring difficulties in diagnosis and management,physicians must pay special attention to it. The fulminant epidemics induced by drug-resistant strains are more likely to occur in neonates, especially those with underlying diseases and immature immunity. Therefore, timely detection, isolation and effective treatment is beneficial to recovery and prognosis. In our study, the overall outcome of all infants was favorable when R.planticola was treated with antibiotic. Pacilli M et al. [12] retrospectively analyzed the relevant literature indicating that no death occurred in newbrons with R.planticola infection, which may be associated to early recognition and effective therapy.

Although the infection time of the four infants were different, all the events occurred in May and June, which may be closely related to the hot and humid summer climate in our region. Koc S et al. [16] determined that the R.planticola isolated from a surface water was resistant to Penicillins and cephalosporins. Multidrug-resistant and multi-metallic resistant strains of R. planticola may also exist in the natural environment [16]. Pan Z et al .[13] found the gene New Delhi Metallo-β-lactamase-1 (blaNDM-1) in multidrug-resistant R. planticola. They think that R. planticola is a possibly underestimated pathogen that contributes to the spread of the blaNDM-1 gene. So,environmental bacteria can easily transfer drug-resistant genes to human pathogens, resulting in the emergence of clinical multi-drug resistant strains [13]. When the R.planticola infection occured, we tried an attempt to identify all possible sources of infections, including sampling and testing the drinking water, condensate pipe water, hand sanitizer, warm box cleaning water and domestic water in the ward. None of R.planticola were determined.

To conclude, R.planticola is also a potential etiologic agent of infections in NICUs, especially challenging infections caused by multidrug-resistant R.planticola strains will probably become a concern for clinicians [5].Herein,pediatricians should not only choose antibiotics according to susceptibility testing, but also timely isolate patients to prevent an outbreak of infection. To the best of our knowledge,there are few literature on neonatal R.planticola infection. Therefore,further pediatric studies will be helpful to understand the clinical characteristics of the R. planticola infections and establish adequate management .

## Data Availability

n/a.

## Abbreviations

rRNA: Ribosomal ribonucleic acid
NICU: Neonatal intensive care unit
PICC: Peripherally inserted central catheter
CRP: C-Reactive protein
PCT: Procalcitonin
ICU: Intensive care unit
